# A case series: thirty-three cases with heterotopic pregnancy after assisted reproductive technology

**DOI:** 10.3389/fmed.2026.1849479

**Published:** 2026-07-14

**Authors:** Xinyang Li, Han Zhang, Qi Xi, Ninglu Yuan, Di Su, Linlin Liu, Zhuo Li, Jiayi Tian

**Affiliations:** Department of Prenatal Diagnosis and Reproductive Medicine Center, The First Hospital of Jilin University, Jilin University, Changchun, China

**Keywords:** ectopic pregnancy, heterotopic pregnancy, intrauterine pregnancy, pregnancy outcome, sac development

## Abstract

**Objectives:**

This study was designed to explore the impacts of clinical features, surgical timing, embryo transfer type, and ectopic pregnancy location on intrauterine pregnancy outcomes among heterotopic pregnancy patients, with the goal of offering evidence-based guidance for clinical management.

**Methods:**

A retrospective analysis was conducted on 33 patients with clinically diagnosed heterotopic pregnancy admitted to our center between June 2019 and September 2025.

**Result:**

This study included 33 patients with heterotopic pregnancy one conceived after ovulation induction and 32 via *in vitro* fertilization (IVF). Final follow-up data showed that 66.67% of patients attained viable ongoing intrauterine gestation or live birth; no fetal structural malformations were observed during the entire follow-up period. Sequential identification of an intrauterine gestational sac, embryo and fetal cardiac activity on ultrasound was associated with progressively higher odds of successful intrauterine pregnancy among patients with heterotopic pregnancy. All three sonographic markers predicted favorable pregnancy outcomes (all *P* < 0.001). Visualized fetal cardiac activity exhibited the strongest predictive value for sustained pregnancy and live birth (OR = 22.00, 95% CI 3.24–149.30, *P* < 0.001). No significant differences in pregnancy outcomes were observed between blastocyst (D5) and fresh embryo transfers compared with D3 cleavage-stage embryos (OR = 0.42, 95% CI 0.08–2.14, *P* = 0.412; OR = 0.67, 95% CI 0.05–8.91, *P* = 1.000, respectively). Cases with better intrauterine embryonic development had significantly higher odds of favorable pregnancy outcomes versus those with poorer intrauterine development (OR = 28.75, 95% CI 2.62–315.41, *P* = 0.003).

**Conclusion:**

Ultrasonic indicators reflecting the development of the intrauterine gestational sac, such as the timing of yolk sac and fetal heartbeat visualization, and crown-rump length, were significantly superior to those of the ectopic gestational sac, indicating a favorable prognosis for the intrauterine pregnancy.

## Introduction

Heterotopic pregnancy (HP) is a pathological condition with multiple gestational sacs implanted at distinct intrauterine and extrauterine sites, a rare subtype of ectopic pregnancy. Classified by ectopic implantation location, it includes intrauterine-tubal HP and variants involving cornua uteri, cervix, cesarean scar, ovary, abdomen, etc. HP is extremely rare in spontaneous pregnancies, with an incidence of one in 30,000 (1). Its incidence has increased with assisted reproductive technology advancement, but true rate remains unclear (2). Increased incidence may be linked to tubal damage, as well as to the transfer of more than a single embryo. Serial plasma human chorionic gonadotrophin (HCG) monitoring represents an important tool for the diagnosis of ectopic pregnancy. Nevertheless, in heterotopic pregnancy, the presence of an intrauterine pregnancy obscures the extrauterine lesion, which compromises its diagnostic value and consequently makes the diagnosis of heterotopic pregnancy particularly arduous. The risk of rupture of the ectopic lesion is positively correlated with the gestational age. Rupture events frequently result in hemorrhagic shock and can be life-threatening in severe instances. In recent years, laparoscopic resection of ectopic gestational lesions has become the main therapeutic strategy for heterotopic pregnancy. However, conservative management may still be an option in selected patients under close ultrasound monitoring. At present, no consensus has been reached regarding the optimal treatment modality, and whether different treatment protocols increase the risk of fetal malformation requires further validation in large sample studies. This retrospective study was designed to explore factors associated with intrauterine embryonic development and to evaluate how differences in developmental status between intrauterine and ectopic gestational sacs at surgery affect subsequent pregnancy outcomes.

## Materials and methods

A total of 33 patients diagnosed with heterotopic pregnancy (HP) by transvaginal sonography (TVS) or surgical pathological examination were enrolled at the Prenatal Diagnosis Center, Reproductive Medicine Center, the First Hospital of Jilin University, during the period from June 2019 to September 2025. Inclusion criteria: HP confirmed by preoperative ultrasonography, intraoperative gestational tissue identification or postoperative pathological report. Ultrasonographic diagnostic criteria: The first ultrasonographic examination was performed at 20–28 days after embryo transfer. A diagnosis was made when an intrauterine gestational sac was visualized, accompanied by any of the following ectopic ultrasonic findings: Mixed, (i) Hypoechoic, or isochoric adnexal mass; (ii) Visualization of a circumferential hyperechoic ectopic gestational sac; (iii) Identification of a yolk sac and/or embryonic bud within the gestational sac, with or without primitive cardiac activity.(3)

Exclusion criteria: (i) Individuals suspected of heterotopic pregnancy on initial transvaginal ultrasound without definitive intraoperative or postoperative pathological confirmation of this diagnosis. (ii) Subjects tentatively suspected of heterotopic pregnancy via ultrasonography at our center with final diagnoses confirmed at outside medical facilities (iii) Cases with fully regressed extrauterine gestational lesions and residual intrauterine gestation on follow-up ultrasound.

This retrospective study was approved by the Institutional Review Board of the First Hospital of Jilin University. Informed consent was waived for anonymous de-identification of clinical data. All relevant data were extracted from the hospital’s inpatient and outpatient registration systems, including hospitalization records and auxiliary examination results of heterotopic pregnancy patients at diagnosis and treatment. As enrollment was restricted to patients with complete accessible clinical archives, the study cohort constituted a convenience sample. Telephone and outpatient clinic visits constituted the primary follow-up modalities. All study procedures were conducted in strict accordance with the Declaration of Helsinki.

Statistical analysis was performed using SPSS 24.0 software. Quantitative data are expressed as mean ± standard deviation (SD). Comparisons between two groups were performed using the independent samples *T*-test. Categorical variables were expressed as numbers and percentages. Between-group comparisons were performed using Fisher’s exact test to reduce potential statistical bias attributable to the limited sample size. All tests were two-sided, with statistical significance set at two-sided *P* < 0.05.

Due to the low prevalence of HP, an a priori sample size calculation was not feasible. To maximize the study sample, we included every consecutive patient with complete medical records who met our eligibility criteria during the predefined study period. Following completion of all statistical tests, we ran *post hoc* power analysis using G*Power 3.1 to determine the statistical discriminative power of this modest-sized cohort.

## Results

### Clinical characteristics and manifestations of the patients

A total of 33 patients were enrolled in this cohort, among whom 14 (42.42%) presented with primary infertility and 19 (57.58%) with secondary infertility. The enrolled patients had a mean maternal age of 33.94 ± 4.41 years, with most subjects being below the threshold of advanced maternal age. The primary cause of infertility in patients undergoing IVF treatment was attributable to female pelvic and tubal factors, accounting for 72.7% of all cases. Eight patients had undergone ipsilateral salpingectomy, one patient was managed with conservative linear salpingostomy. Nineteen patients had a history of hysteroscopic evaluation or adhesiolysis. Additionally, 14 patients had previously undergone salpingectomy or ovarian cystectomy involving the mesosalpingeal region, of whom 10 had undergone concurrent uterine and adnexal procedures. Thirteen patients were asymptomatic on presentation and were referred exclusively for abnormal sonographic findings. And 20 patients developed symptoms during sonographic surveillance, most commonly abdominal pain, vaginal bleeding, or both. Notably, three patients developed hemorrhagic shock during the clinical course as a critical adverse event (Table 1).

**TABLE 1:** Baseline characteristics and pregnancy outcomes of patients.

Characteristics	*n* (%)
Age, year; mean ± SD (range)	33.94 ± 4.41
Causes of infertility, n (%)	
primary infertility	14 (42.4)
secondary infertility	19 (57.6)
History of surgery, n (%)	
History of adnexal surgery	14 (42.4)
History of uterine surgery	9 (57.6)
History of other surgical procedures	7 (9.4)
No surgery	6 (18.2)
Previous ectopic pregnancy, *n* (%)	9 (57.6)
Site of ectopic pregnancy, n (%)	
Tubal	26 (78.8)
- Non-interstitial tubal	15 (45.5)
-Interstitial	11 (33.3)
Cervical pregnancy	3 (9.1)
Ovarian	1 (3.0)
Clinical features, n (%)	
Hypovolemic shock	3 (9.1)
Abdominal pain or/and vaginal bleeding	20 (60.6)
Asymptomatic	13 (39.4)
Hypovolemic shock	3 (9.1)

### Embryo selection

Thirty-two cases received double embryo transfer, while one case achieved spontaneous conception after ovulation induction. The frozen cleavage-stage embryo transfer group yielded the highest clinical pregnancy rate (75%), which was higher than that in the blastocyst-stage transfer group (55.6%) and the fresh embryo transfer group (66.7%). *Post hoc* power analysis was conducted in G*Power 3.1. Based on the observed pregnancy rates, actual group sample sizes and a two-sided α of 0.05 for independent binomial proportions, the statistical power was calculated as 0.054. Overall differences among the three groups were assessed using a Fisher exact test (*P* = 0.642). No significant intergroup difference was identified (*P* > 0.05), as summarized in Table 2.

**TABLE 2:** Embryo transfer and pregnancy outcomes.

Type of transferred embryo	Favorable outcome group, *n* (%)	Adverse outcome group, *n* (%)	Test statistic	*P*	OR (95% CI)
Cleavage-stage embryo (D3)	15 (75)	5 (25)	Ref.	Ref.	Ref.
Blastocyst (D5)	5 (55.6)	4 (44.4)	FET	0.412	0.42 (0.08–2.14)
Fresh embryo	2 (66.7)	1 (33.3)	FET	1.000	0.67 (0.05–8.91)

All pairwise comparisons adopted Fisher’s exact test using D3 cleavage-stage embryo as the reference.

### Ectopic pregnancy site and outcomes

**FIGURE 1:**
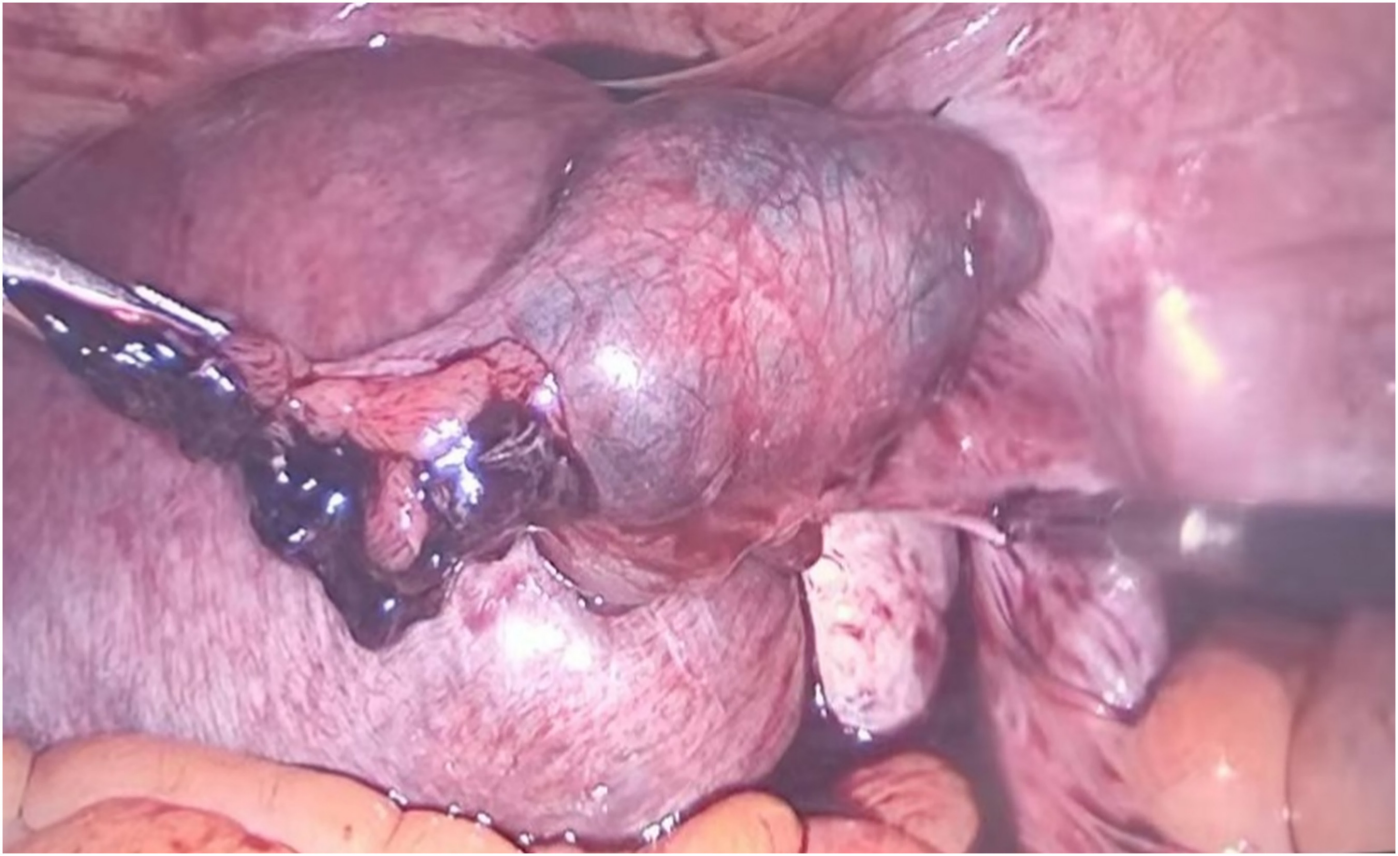
Intraoperative laparoscopic views of ruptured ampullary ectopic pregnancy. A distended ruptured ampullary fallopian tube with pelvic hemoperitoneum and adherent blood clots.

**FIGURE 2:**
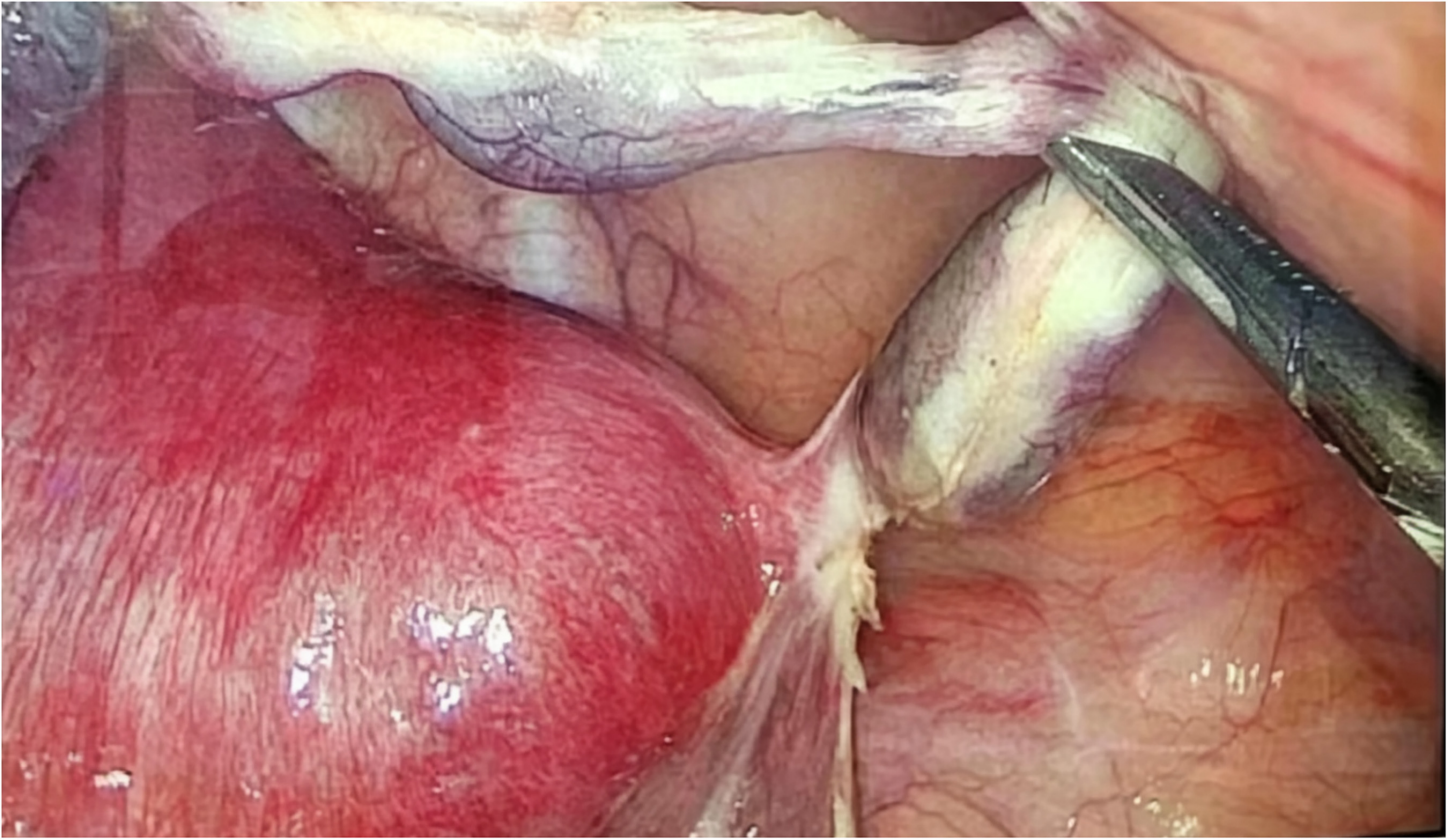
Intraoperative laparoscopic view of interstitial tubal pregnancy. Laparoscopic atraumatic grasper retract adjacent tissue, exposing the gestational sac in the interstitial tubal segment.

Treatment was determined by the location of the ectopic pregnancy. Given the life-threatening risk of cervical ectopic pregnancy, all three patients underwent ultrasound-guided uterine curettage. An ovarian pregnancy was treated with ovarian pregnancy tissue resection, which yielded favorable reproductive outcomes. Among the 26 cases of tubal pregnancy, the intrauterine pregnancy survival rate was 86.7% in patients with non-interstitial tubal ectopic pregnancy after treatment, compared with 72.7% in those with interstitial tubal pregnancy. For patients with cornual and other heterotopic ectopic pregnancies, no persistent intrauterine pregnancy was detected. Figure 1 shows intraoperative laparoscopic images of lesions from non-interstitial tubal pregnancy, and Figure 2 presents laparoscopic intraoperative findings of interstitial tubal pregnancy.

### Ultrasound detection time of intrauterine and ectopic gestational focus

Among the 32 patients with intrauterine gestational sacs detected 20–28 days after embryo transfer, ectopic lesions were identified concurrently in 29 cases. In the remaining three patients, the detection of ectopic lesions was delayed by 2, 14, and 20 days, respectively, compared with the identification of intrauterine pregnancy. At the time of ultrasonic diagnosis of heterotopic pregnancy, embryonic cardiac activity was observed in the intrauterine gestation in 23 cases and in the ectopic lesion in two cases, with simultaneous detection of fetal heartbeat in both intrauterine and ectopic sites in one case. No embryonic cardiac activity was detected in the intrauterine pregnancy of another eight patients, and in one of these cases, the intrauterine lesion was identified 2 days earlier than the ectopic lesion.

**FIGURE 3:**
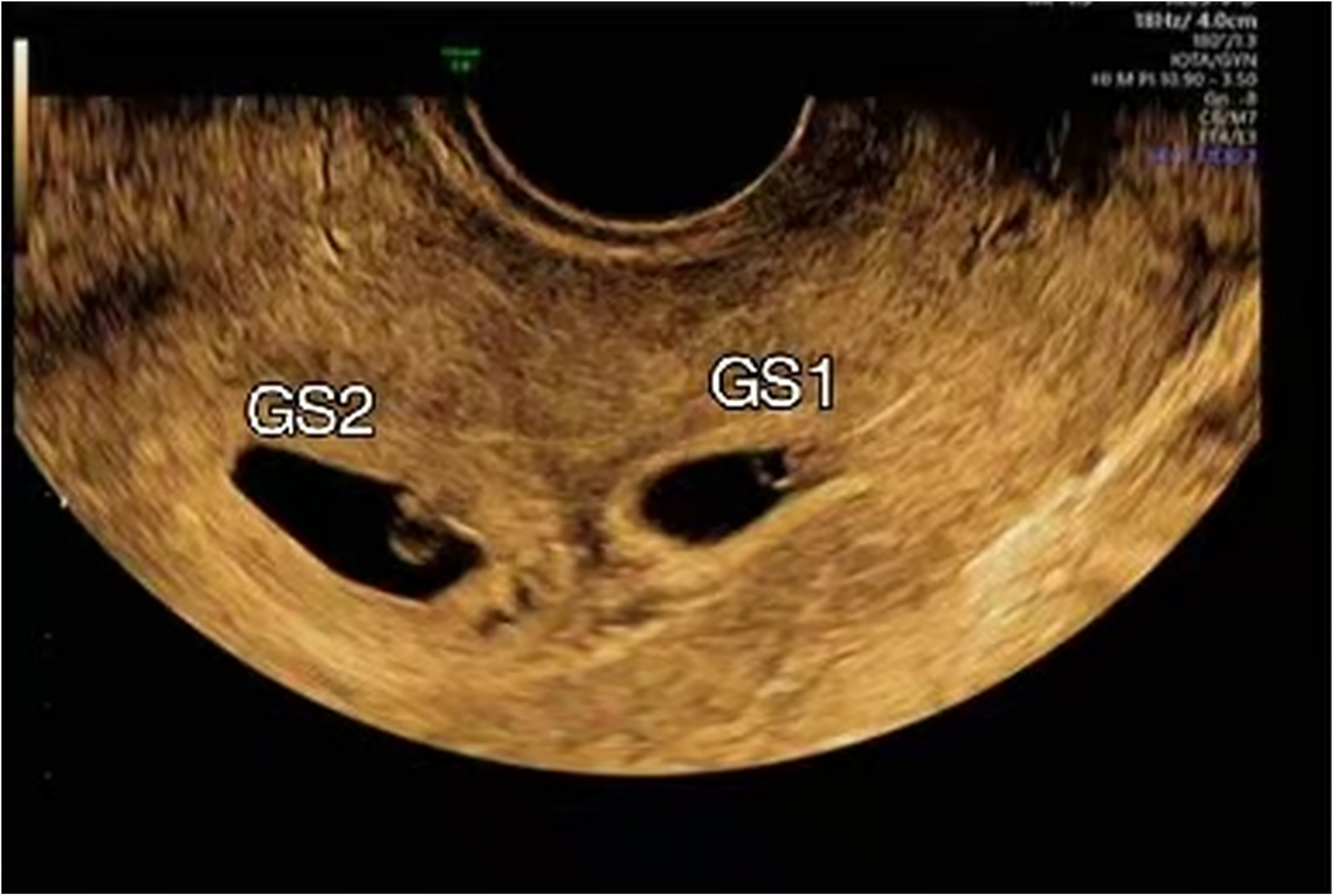
GS1, gestational sac located within the cervix; GS2, intrauterine gestational sac.

**FIGURE 4:**
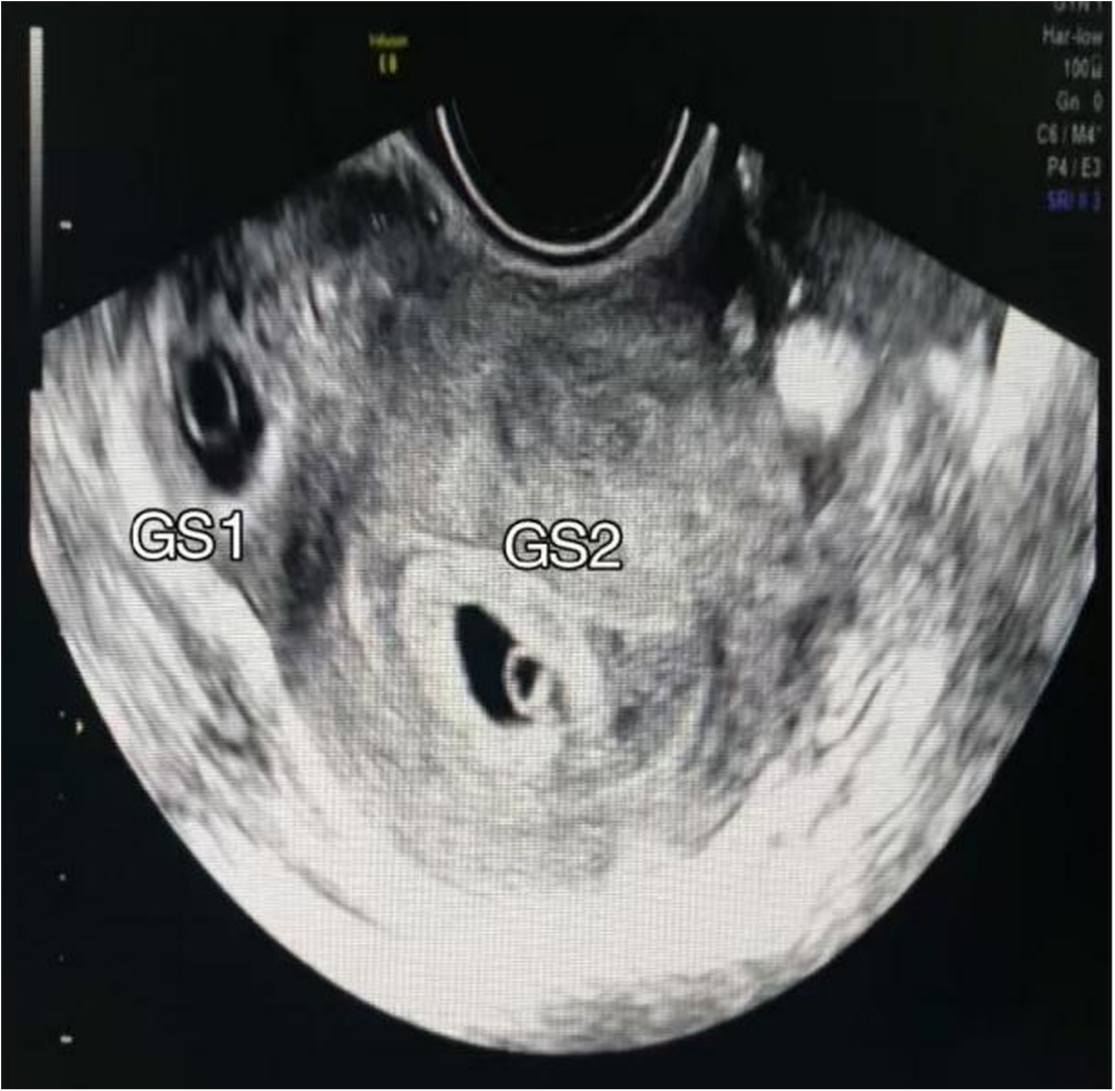
GS1, gestational sac within the interstitial segment of the fallopian tube; GS2, intrauterine gestational sac.

Based on the above ultrasound evaluation criteria, 27 cases in this study presented with better intrauterine pregnancy development than ectopic pregnancy. Figure 3 presents typical transvaginal ultrasonographic findings of cervical heterotopic gestational sacs, and Figure 4 shows corresponding ultrasonographic features of gestational sacs located in the interstitial segment of the fallopian tube. *Post hoc* statistical power analysis was performed for subgroup comparisons using G*Power 3.1 software. Two-tailed *Z*-tests for independent binomial proportions were adopted with a significance level of α = 0.05. The actual sample sizes of enrolled cohorts and clinically observed pregnancy success rates were included for calculation, yielding an achieved statistical power of 0.959. Fisher’s exact test was used to examine associations between intrauterine sonographic findings and clinical pregnancy success. Visible gestational sac, embryo, and fetal heart activity were each linked to greater odds of favorable pregnancy results; corresponding odds ratios and 95% confidence intervals are listed in Table 3. Compared with patients with inferior intrauterine embryonic development, those with superior intrauterine development had significantly higher odds of favorable pregnancy outcomes, as shown in Table 4.

**TABLE 3:** Preoperative ultrasound findings.

Intrauterine ultrasound findings	Total, *n*	Successful pregnancy, *n*	Success rate (%)	OR (95% CI)	*P*
Visible gestational sac	27	23	69.7	4.50 (2.22–9.11)	<0.001
Visible embryo	23	21	63.6	11.50 (3.06–43.23)	<0.001
Visible heartbeat	22	21	63.6	22.00 (3.24–149.30)	<0.001

Patients without the corresponding intrauterine structure served as the reference group (OR = 1.00). OR indicates the odds of successful pregnancy.

**TABLE 4:** Embryonic development and pregnancy outcomes.

Intrauterine and extrauterine development	Total, *n*	Favorable outcome, *n* (%)	Adverse outcome, *n* (%)	OR (95% CI)	*P*
Intrauterine development superior to extrauterine	27	23 (69.7)	4 (12.1)	28.75 (2.62–315.41)	0.003
Intrauterine development inferior to extrauterine	6	1 (3.0)	5 (15.2)	1	–

The parallel inferior-development group served as reference (OR = 1.00). OR, odds of favorable pregnancy outcomes.

## Discussion

Early clinical manifestations of HP are atypical. Abdominal pain or vaginal bleeding can also occur in patients with isolated intrauterine pregnancy or ectopic pregnancy. Furthermore, such symptoms can be physiological following embryo transfer, thereby contributing to significant delays or missed diagnoses of heterotopic pregnancy in clinical practice particularly when serum HCG levels are within the normal range for pregnancy and an intrauterine pregnancy has been clearly identified, which may lower clinical vigilance.

In our study, 39.39% of patients were asymptomatic, with only extrauterine abnormal echogenicity raising high suspicion of heterotopic pregnancy. Most cases of HP are diagnosed during surgery. Although ultrasound allows for early diagnosis of HP in some cases, enlarged ovaries following ovulation induction, as well as structural anomalies of the ovaries and fallopian tubes in infertile patients, substantially increase the difficulty of ultrasonographic diagnosis. In addition, some patients cannot attend follow-up visit for various reasons, which prevents serial ultrasound monitoring and may eventually lead to severe complications such as ectopic pregnancy rupture and hemorrhagic shock. According to the existing literature, the ultrasonographic diagnostic rate of HP is only 20%–50% (3).

In this study, six patients presented with obvious peritoneal irritation such as marked tenderness on admission. Three patients already showed hemorrhagic shock, including altered mental status and decreased blood pressure.

Ultrasound monitoring after embryo transfer is an ongoing, dynamic assessment. Wu suggested that the first ultrasound scan is usually performed at 4–5 weeks after embryo transfer, with a follow-up examination scheduled 2 weeks later once intrauterine pregnancy with cardiac activity has been confirmed and ultrasound evaluation should be performed promptly in patients with vaginal bleeding or abdominal pain(4). In our reproductive center, patients undergoing assisted reproductive technology routinely undergo their first ultrasound at 4 weeks after embryo transfer. The primary purpose is to confirm the intrauterine location of the gestational sac, assess the number and basic morphology of the sacs, and guide adjustments to subsequent treatment. A study discovered that about 70% of HP cases are diagnosed between 5 and 8 weeks of gestation, 20% between 9 and 10 weeks, and 10% after 11 weeks (5).

Shang.et al. declared that patients with an intrauterine gestational sac without cardiac activity at the time of HP diagnosis had a higher miscarriage rate than those with cardiac activity. Moreover, the presence of cardiac activity in the intrauterine gestational sac at diagnosis was identified as an independent predictor of pregnancy outcome (6). In this study, intrauterine gestational sacs and ectopic pregnancies were detectable in most patients as early as 4–6 weeks after embryo transfer. The developmental status of intrauterine and extrauterine embryos at this stage including the timing of gestational sac appearance and the presence of fetal buds and primitive cardiac activity carries important implications for clinical management. Combined with the above findings, this study further analyzed the dynamic ultrasound findings of the patients. Among the 27 patients in this study, intrauterine gestational sacs or fetal buds emerged earlier and exhibited better development than extrauterine lesions, and these patients had a notably higher rate of final live birth or ongoing pregnancy following treatment. This demonstrates that dynamic tracking of both pregnancy location and embryonic growth status enables not only early identification of heterotopic pregnancy but also effective evaluation of pregnancy prognosis.

This study analyzed the developmental indicators of pregnancy structures in 33 patients during their first ultrasound examination, including gestational sac size at the initial detection of intrauterine pregnancy, yolk sac formation status, fetal bud length, presence or absence of fetal cardiac activity, and the initial appearance time of extrauterine gestational sacs. We further demonstrated that earlier visualization of fetal buds and cardiac activity in the intrauterine gestational sac, coupled with better intrauterine embryonic development relative to the extrauterine sac, serves as the key determinant of pregnancy outcome, regardless of the timing of surgery.

Currently, the pathogenesis of heterotopic pregnancy remains unclear. Some scholars believe that its etiology is highly similar to that of ectopic pregnancy. *In vitro* fertilization (IVF) has been identified as a major risk factor for high-risk pregnancies (HP), particularly following non-donor cycle transfers or multiple embryo transfers in assisted reproductive technologies (ART). According to the guidelines of European Society of Human Reproduction and Embryology (ESHRE), single embryo transfer is recommended to achieve better pregnancy outcomes. And the specific transfer protocol should be tailored based on factors such as embryo quality, developmental stage, maternal age, ovarian response, and treatment protocol. The guidelines do not support the transfer of more than two embryos.

In isolated ectopic pregnancy, the most frequent site of extrauterine implantation is the ampullary portion of the fallopian tube, followed by the isthmus and fimbriae. Interstitial pregnancy is comparatively rare. However, among HP patients in this study, the proportion of cases complicated by interstitial tubal pregnancy was as high as 30.30%, which was significantly higher than the incidence of interstitial pregnancy reported in previous studies of isolated ectopic pregnancy. This suggests that even with a definite intrauterine gestational sac on ultrasound, clinicians must maintain a high index of suspicion for concomitant interstitial tubal pregnancy.

This may be explained by the interstitial portion of the fallopian tube forming the junction between the tube and the uterine cavity. As this segment lies within the myometrium with a thick muscular layer and abundant blood supply, when embryos migrate toward an abnormal implantation site, they may become arrested and implant at this location. In patients with a history of pelvic inflammatory disease, even without complete tubal obstruction impaired tubal peristalsis can predispose the fertilized ovum to implant at this location (7). Other possible risk factors include previous ectopic pregnancy, prior abortion, and poor pelvic conditions due to pelvic infection (8). In our study, 19 ART patients (57.6%) had a prior history of ectopic pregnancy or abortion, further confirming an association between these factors and heterotopic pregnancy (HP). Therefore, clinicians should maintain a high index of suspicion for the development of heterotopic pregnancy in women with these high-risk factors.

Common treatment methods for HP include laparoscopic fetal reduction, uterine curettage, medical treatment and expectant management, surgical treatment is the preferred approach. But for cornual pregnancy, complete resection of the ectopic lesion cannot be achieved while maintaining the viability of the intrauterine pregnancy. In our study, all patients with combined intrauterine and cornual pregnancy underwent either curettage or medical abortion for termination.

A study demonstrated that patients treated with ultrasound-guided embryo aspiration had improved pregnancy outcomes. In contrast, those in the expectant management group faced a high risk of ectopic pregnancy lesion rupture. Surgical treatment can completely remove ectopic lesions, but the abortion rate of intrauterine pregnancy is relatively high after the operation (9). In this study, for patients who received expectant management, no abnormalities were observed in the development of their intrauterine pregnancy. There remains controversy regarding which treatment method is more advantageous; early diagnosis and individualized treatment are the keys to the management of HP patients.

In recent years, numerous studies have indicated that different types of transferred embryos can lead to varied maternal estrogen levels, thereby raising the risk of ectopic pregnancy. Currently, existing evidence suggests that the pregnancy success rate of frozen-thawed embryo transfer is higher than that of fresh embryo transfer, and the risk of complications such as ovarian hyperstimulation is lower (10). Besides, among IVF patients with at least four embryos retrieved and a good prognostic profile, cumulative live birth rates were comparable between blastocyst transfer and cleavage-stage transfer. While blastocyst culture facilitates the selection of embryos with superior developmental competence and achieves better synchrony with the endometrial implantation window, cleavage-stage protocols yield a larger number of cryopreserved embryos. Repeated frozen-thawed transfers can offset the suboptimal performance of fresh cycles, leading to equivalent overall reproductive outcomes between the two groups (11). But the research conclusions on which type of embryo transfer can reduce the risk of ectopic pregnancy remain inconsistent. Zhang.et al. reported that the risk of ectopic pregnancy was lower in frozen-thawed embryo transfer cycles than in fresh embryo transfer cycles. In addition, blastocyst transfer was associated with a further reduction in the rate of ectopic pregnancy during frozen-thawed embryo transfer (12). Hu et al. observed comparable ectopic pregnancy rates following fresh and frozen-thawed embryo transfer (13). This study included patients with heterotopic pregnancy who received fresh embryo transfer or frozen-thawed embryo transfer with varying *in vitro* culture periods. Appropriate surgical or conservative management yields favorable gestational outcomes in women with heterotopic pregnancy after frozen embryo transfer. This may be attributed to excessive estrogen exposure induced by ovarian stimulation in fresh cycles, which leads to asynchrony between embryonic development and endometrial maturation and thereby impairs endometrial receptivity, which is detrimental to the progression of intrauterine pregnancy (10). Regarding the impact of different types of embryo transfer on the incidence of ectopic pregnancy, the meta-analysis encompassing 22 studies with a total of 143,643 pregnancies found that day-5 transfer decreased ectopic pregnancy risk versus day-3 transfer, with the protective effect most prominent in frozen–thawed cycles. Compared with day 3 embryo transfer, day 5 blastocyst transfer reduces ectopic pregnancy risk by shortening embryo migration time and selecting viable embryos. The effect becomes stronger in frozen-thawed cycles, as excessive estrogen in fresh cycles may impair tubal function and weaken this protective effect (14). Another meta-analysis indicates that there is no clear correlation between endometrial thickness and the incidence of ectopic pregnancy. A thin endometrium cannot serve as a high-risk predictor for ectopic pregnancy. As a valid indicator reflecting overall endometrial receptivity, endometrial thickness mainly affects embryo implantation, pregnancy maintenance and live birth outcomes (15). However, it remains unclear whether the type of embryo transfer affects pregnancy outcomes in patients with heterotopic pregnancy. In our study, 66.7% of patients diagnosed with HP after fresh embryo transfer achieved a live birth. For patients who developed HP after blastocyst transfer, this live birth rate was 55.6%. In patients receiving day 3 embryo transfer, the live birth rate was 75% (15/20). Extended *in vitro* culture for blastocyst formation acts as a natural embryo screening mechanism, yet it may impair embryonic developmental competence. By comparison, Day 3 cleavage-stage embryos experience shorter culture duration and retain stronger developmental potential, which is conducive to subsequent intrauterine implantation and fetal development. But statistical analyses revealed no intergroup differences. The small number of eligible studies reduced the reliability of our statistical assessment, rendering the results of limited practical value.

## Strengths and limitations

The present study has a number of inherent limitations. First, this retrospective analysis enrolled merely 33 eligible patients, who were subsequently stratified into several smaller subgroups for subgroup comparisons. Given the rarity of the investigated disorder and the finite pool of retrievable historical medical records, we were unable to conduct a priori sample size estimation or prespecified power calculation prior to data collection. As a result, the study possessed limited statistical power to identify modest between-group differences, which may compromise both the interpretability of statistical comparisons and the external validity of our observations.

Second, all participants were recruited from a single institution according to the availability of archived clinical data, forming a convenience sample. Large-scale, multicenter cohorts are therefore warranted to replicate and verify the present findings in future investigations.

## Conclusion

Overall, HP is extremely rare, however, its incidence has risen substantially in recent years, attributed to the widespread use of assisted reproductive technology and an increasing number of patients with a history of pelvic and intrauterine inflammation. Dynamic ultrasound monitoring of the development of intrauterine and ectopic gestational sacs is crucial for evaluating the outcome of intrauterine pregnancy. Laparoscopy is the main treatment modality, and in most instances, the intrauterine pregnancy can proceed uneventfully to term. Meanwhile, the treatment plan should be improved by integrating the patient’s medical history, the type of embryo transfer, and the location of ectopic pregnancy to further optimize the pregnancy outcome.

## Data Availability

The original contributions presented in this study are included in this article/supplementary material, further inquiries can be directed to the corresponding author.
